# Vasopressin in Sepsis and Other Shock States: State of the Art

**DOI:** 10.3390/jpm13111548

**Published:** 2023-10-29

**Authors:** Raquel García-Álvarez, Rafael Arboleda-Salazar

**Affiliations:** Department of Anesthesiology and Surgical Intensive Care, University Hospital 12 de Octubre, 28022 Madrid, Spain

**Keywords:** vasopressin, septic shock, vasodilatory shock, vasoplegia

## Abstract

This review of the use of vasopressin aims to be comprehensive and highly practical, based on the available scientific evidence and our extensive clinical experience with the drug. It summarizes controversies about vasopressin use in septic shock and other vasodilatory states. Vasopressin is a natural hormone with powerful vasoconstrictive effects and is responsible for the regulation of plasma osmolality by maintaining fluid homeostasis. Septic shock is defined by the need for vasopressors to correct hypotension and lactic acidosis secondary to infection, with a high mortality rate. The Surviving Sepsis Campaign guidelines recommend vasopressin as a second-line vasopressor, added to norepinephrine. However, these guidelines do not address specific debates surrounding the use of vasopressin in real-world clinical practice.

## 1. Introduction

Arginine vasopressin (AVP) is a non-catecholamine hormone produced in the hypothalamus and released into the circulation through the posterior pituitary gland. It was isolated it in 1895 [1] from the extract of the posterior pituitary. After the identification of the molecule, its potent antidiuretic effects and its benefits in diabetes insipidus (DI) led it to be renamed as antidiuretic hormone (ADH) [2]. Du Vigneaud synthesized AVP and described its structure in early 1950s, earning him the Nobel Prize in 1955 [3]. The AVP used as a therapeutic agent is of synthetic origin and is structurally identical to the human peptide hormone. Until about 30 years ago, AVP was employed to manage polyuria in patients with DI [4] and minimize blood loss in gastrointestinal bleeding [5]. It was not until the early 1990s that its clinical vasopressor effect began to be used, establishing the drug as potentially useful in the treatment of vasodilatory shock [6].

Throughout this review, we will try to explain the rationales for using AVP in septic shock, based on four pillars:

- AVP deficiency present in septic shock [7].
- As a multimodal strategy for the sparing of catecholamines.
- Its potential nephroprotective effect.
- Early onset of AVP infusion.

In addition, we will review the scientific evidence for its use in other catecholamine-resistant shock states.

## 2. Physiology

### 2.1. Synthesis and Release

Arginine vasopressin is a little peptide composed of nine amino acids, with arginine occupying the eighth position [8]. It is produced by magnocellular neurosecretory neurons located in the anterior hypothalamus that directly function as osmoreceptors. AVP subsequently migrates (as a prohormone) along the supraoptic-hypophyseal tract to the posterior pituitary gland (neurohypophysis), where it is stored in vesicles and is released into the circulation in response to appropriate stimuli. The most important stimuli producing AVP release are mainly an increase in plasma osmolarity and/or decreased blood volume [9]. Its half-life is 5–15 min [10].

### 2.2. AVP Receptors and Signal Transduction

**Figure 1:**
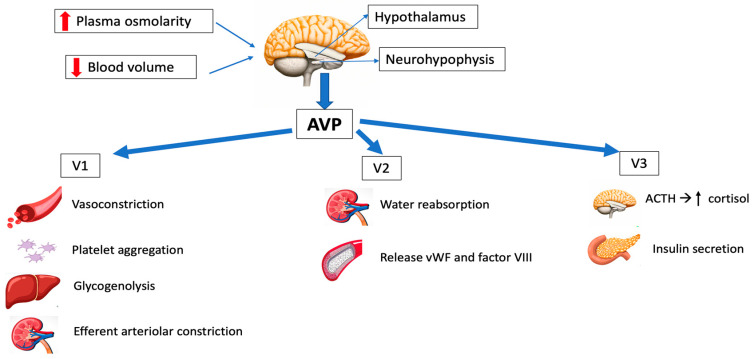
Schematic representation of the main physiological effects of vasopressin. AVP: arginine vasopressin; ACTH: adrenocorticotropic hormone; vWF: von Willebrand factor.

Three different types of AVP receptors have been identified (Figure 1): V1 (previously known as V1a, mainly vascular), V2 (mainly renal) and V3 (previously V1b, mainly central). Its location and function are summarized in Table 1.

V1 receptors are mainly expressed on vascular smooth muscle, and their activation leads to vasoconstriction. Other effects of V1 stimulation include the endothelial release of nitric oxide (NO), which causes vasodilation of the coronary and pulmonary vessels [11,12] and platelet aggregation.

V2 receptors are found in the distal tubal and collecting ducts, regulating the antidiuretic effects of AVP, and on vascular endothelium, releasing coagulation factor VIII and von Willebrand factor (vWF), which are important for blood clot formation on bleeding [13].

V3 receptors are found in the anterior pituitary, inducing the secretion of adrenocorticotropic hormone (ACTH), and in the pancreas, resulting in insulin secretion [13,14].

### 2.3. Physiological Functions

AVP has a significant role in osmoregulation, cardiovascular stability and overall homeostasis. Additionally, it acts as a corticotropin secretagogue and has an impact on cognition, learning and memory. However, since AVP does not go through the blood–brain barrier, its central nervous system effects are not relevant in the case of intravenous administration of the drug.

- Osmoregulation

AVP maintains plasma osmolality between 275 and 290 mOsm/kg H_2_O. The most important stimuli of AVP release are an increase in osmolarity or decreased blood volume [9].

DI is caused by a lack of AVP effect. This can be of renal origin (mutations of the V2 receptor) [15] or central origin, with reduced vasopressin release (idiopathic or secondary to brain tumors, brain ischemia or head injuries) [16]. The osmoregulatory functions of AVP can be replaced with a synthetic selective V2 receptor agonist known as desmopressin (DDAVP) [16].

- Cardiovascular control

The maintenance of arterial blood pressure involves the interaction of sympathetic, renin–angiotensin and AVP systems. Under physiological conditions, the role of AVP in regulating arterial blood pressure is minimal, with the influence of vasopressin on vasomotor tone being minimal in healthy subjects. However, if the two other systems are compromised, AVP can play a more significant role, mainly in clinical scenarios of relative vasopressin deficiency such as sepsis and other vasodilatory shock states [17].

- Corticotropin secretion

AVP induces corticotropic axis stimulation (increase in ACTH and cortisol) via the V3 receptors [18].

- Hemostasis

AVP promotes blood clotting, with the release of coagulation factor VIII and von Willebrand factor (vWF) [12]. Desmopressin (DDAVP) is commonly used for bleeding disorders due to having fewer side effects than AVP. In perioperative situations, patients with mild hemophilia A, type 1 von Willebrand disease or congenital or acquired platelet disorders could find advantages in the hemostatic properties of DDAVP [19].

## 3. Pharmacology

AVP requires parenteral administration, since trypsin rapidly hydrolyzes the molecule. It is not protein-bound, and the plasma half-life is 5–15 min, so continuous infusion is necessary. The clearance of AVP is mainly mediated by renal and liver vasopressinases [7,13], and a little part is eliminated in urine without undergoing changes. Normal plasma concentrations are less than 4 pg/mL.

Throughout the years, many efforts have been made to modify AVP and develop analogs with different pharmacological characteristics that could overcome its limitations [13,20]. These analogs involve the alteration of one or more amino acids in the sequence of the molecule, aiming for longer half-lives and better receptor selectivity (Table 1):

- Arginine vasopressin (AVP) acts on the V1, V2 and V3 receptors, and has been employed in the management of refractory vasodilatory hypotension, cardiac arrest and septic shock. It has the great advantage of having a short half-life, so the dose can be easily titrated.
- Desmopressin acetate (DDAVP) is a synthetic agonist with V2 receptor specificity and was first used in management of central diabetes insipidus. By directly affecting the endothelial V2 receptors, DDAVP also raises the plasma factor VIII and vWF concentrations in healthy subjects.
- Terlipressin (TP) has a greater selectivity for the V1 receptor than AVP. It is a prodrug of AVP and undergoes metabolism by exopeptidases to yield the active metabolite lysine vasopressin in the circulation, producing a “slow release” effect and affording a longer biological half-life (6 h). Terlipressin has been used as treatment for bleeding gastric second to esophageal varices, portal hypertension and septic shock. The drug increases blood pressure and improves the outcomes of hepatorenal syndrome (contracting the mesenteric arteries, resulting in decreased portal venous inflow and subsequently lowering portal pressure) [5]. The latest Surviving Sepsis Campaign (SSC) recommendations [21] do not advise its use in patients with septic shock, due to greater undesirable effects (more serious adverse events than NE, especially digital ischemia) [22,23].
- Selepressin, another synthetic vasopressin analog, is a short-acting selective V1 receptor agonist. It may present benefits compared to AVP due its ability to induce pure vasoconstriction; it also has reduced antidiuretic effects, a lower risk of thrombotic complications (because of reduced release of vWF) and affords superior protection from increased permeability. However, recently, a trial was stopped due to futility criteria, because no difference was observed in vasopressor- and ventilator-free days [24]. The drug is currently not approved for clinical use.
- Ornipressin exhibits a particular affinity for V1 receptors, thus mimicking the vascular effects of AVP. It has been reported to reduce blood loss during laparoscopic myomectomy [25] and proved useful in cirrhosis with hepatorenal syndrome [26].

## 4. Vasopressin in Septic Shock

Septic shock is the most frequent cause of vasodilatory shock. In 2016, the Third International Consensus Definition for Sepsis and Septic Shock (Sepsis-3) defined sepsis as life-threatening organ dysfunction resulting from dysregulated host responses to infection. Septic shock was defined as a subgroup of sepsis in which circulatory and cellular metabolic abnormalities are severe enough to significantly elevate the risk of mortality: despite adequate fluid resuscitation, patients experience hypotension requiring the use of vasopressors and have a raised serum lactate concentration of over 2 mmol/L [27]. Sepsis mortality remains higher (25–30%) [28], and even 40–50% when shock is present [29].

The Surviving Sepsis Campaign (SSC) [21] is a global initiative aimed at improving the management of sepsis. The campaign was launched in 2002 by the Society of Critical Care Medicine and the European Society of Intensive Care Medicine. The SSC initially focused on creating guidelines for the treatment of sepsis, which were first published in 2004 and were updated in 2008, 2012, 2016 and 2021. These guidelines have become widely accepted as the standard of care for sepsis, to be undertaken as a medical emergency. The SSC guidelines provide evidence-based recommendations for the management of septic shock which include source control, antibiotic therapy, fluid resuscitation, vasopressor therapy, supportive care and monitoring and follow-up.

**Figure 2:**
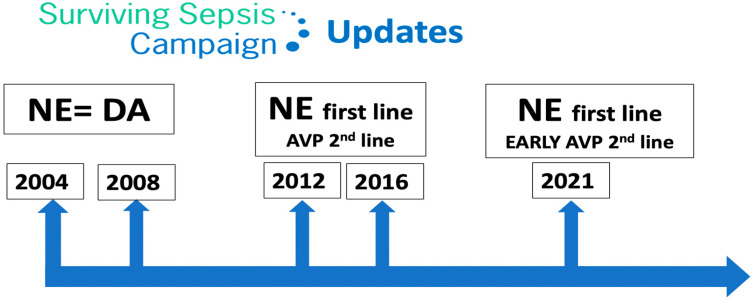
Surviving Sepsis Campaign updates. NE: norepinephrine; DA: dopamine; AVP: arginine vasopressin.

Regarding vasopressor therapy, since 2012 the SSC recommended NE as the first-line vasopressor agent (it previously recommended either NE or dopamine) and suggested adding AVP. However, since 2021 the SSC suggests adding AVP early [21], rather than increasing the NE dose (Figure 2).

The authors of the SSC recognize that certain evidence implies that AVP might be superior to NE in terms of clinical outcomes. However, due to its higher costs and lesser availability, they consider NE as a first-line agent instead of AVP. A recent Cochrane review found that there was insufficient mortality-related evidence to consider any vasopressor as being superior to others [30]. NE is the first-line vasopressor in septic shock because it has been found to be superior to dopamine and equivalent to AVP and epinephrine in randomized controlled trials (RCTs) [31,32,33,34,35,36] (Table 2).

### 4.1. Pathophysiology of Peripheral Vasodilation in Septic Shock

The pathophysiological basis of the septic condition is complex. It is unclear why some patients generate a productive immune response to fight infection, while others deteriorate into a dysregulated state [37]. Traditionally, sepsis was considered to be an extensive, systemic proinflammatory reaction to infection, followed by a phase of immunosuppression marked by anergy, lymphopenia and secondary infections [38,39]. Newer studies propose that the proinflammatory and immunosuppression [40] phases might occur simultaneously, with the magnitude of both responses being influenced by multiple factors of both the host and the pathogen [39,41,42,43].

The main characteristic of septic shock is hypotension [44], and while cardiac dysfunction and hypovolemia may play a role in it, the primary underlying mechanism is peripheral vasodilation [45]. Several neurohormonal responses are triggered by sepsis, including sympathoadrenal activity, the renin–angiotensin system and AVP, which leads to significant peripheral vasoconstriction. However, the vascular smooth muscle shows decreased responsiveness to adrenergic vasoconstrictors, which may cause the accompanying hypotension to prove unresponsive to conventional catecholamine therapy [5].

The primary mechanisms responsible for vasodilation in sepsis are twofold: prostacyclin synthesis and increased nitric oxide (NO) [46]. Other proposed mechanisms that may be targets for future therapies are overproduction of adrenomedullin (a vasodilating hormone and cardiac depressant) and activation of the transient receptor potential vanilloid type 4 (TRPV4) channels [47].

The impact of AVP on cardiac output is controversial. A non-randomized clinical study reported an 11% reduction in cardiac output [48]; conversely, a randomized study with a limited sample size failed to confirm this [49]: cardiac output was similar or even higher in patients receiving AVP in addition to NE [50]. It is important to note that the use of AVP showed non-significant differences in serum troponin levels or electrocardiographic patterns in a randomized controlled trial comparing NE versus AVP in septic shock patients [51].

### 4.2. Treatment of Vasodilation in Septic Shock

The cornerstone of the hemodynamic treatment of septic shock involves fluid resuscitation followed by the use of vasopressors when fluids alone are insufficient to achieve the desired target perfusion. These vasopressors can be classified into two types: pure vasoconstrictors and catecholamines (Table 3). Pure non-catecholamine vasoconstrictors exert an exclusive effect upon vessels and have no direct cardiac inotropic effect. Examples of pure vasoconstrictors include AVP, phenylephrine and angiotensin II. These drugs offer significant benefit as they do not cause direct cardiac toxicity [52]. In addition to inducing vasoconstriction, catecholamines (such as NE or epinephrine) are used as inotropes due to their ability to activate beta receptors. This activation leads to an increase in cardiac output and heart rate, which can be advantageous. However, the risk of cardiac toxicity also increases, particularly at higher doses.

Failure to respond satisfactorily to catecholamines is generally associated with metabolic abnormalities such as a systemic inflammatory response or acidosis, which can lead to alpha receptor desensitization. These abnormalities can disrupt nitric oxide metabolism and increase the accumulation of reactive oxygen species. Such non-responsiveness could also be linked to absolute or relative deficiencies of corticosteroids [53].

### 4.3. Rationale for AVP Use in Septic Shock

**Figure 3:**
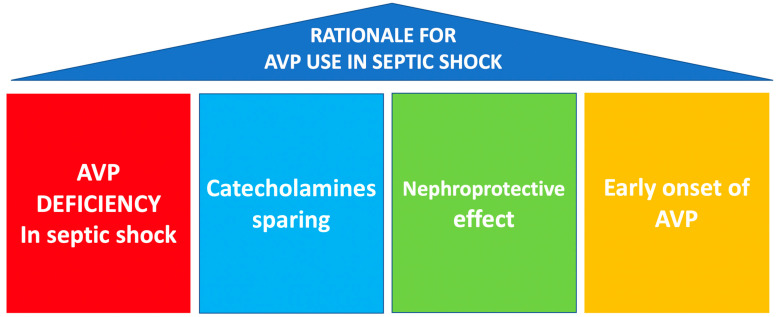
Rationale for AVP use in septic shock.

As we have mentioned before, the rationale for using AVP in septic shock is based on four pillars (Figure 3):

- AVP deficiency present in septic shock [6].
- As a multimodal strategy for the sparing of catecholamines.
- Its potential nephroprotective effect.
- Early onset of AVP infusion.

#### 4.3.1. AVP Deficiency in Septic Shock

In septic shock, AVP concentration exhibits biphasic changes, with elevated concentrations in the early phase to maintain organ perfusion, though as the shock state progresses, these concentrations decrease: vasopressin concentrations initially increase markedly because of hypotension and then decline progressively over 72 h to levels that are too low [6] compared with similarly hypotensive patients with cardiogenic shock [54]. This finding has been called “relative vasopressin deficiency”, because in the presence of hypotension, AVP would be expected to be high; it is a result of the depletion of natural stores and impaired synthesis and secretion of AVP. Landry et al. [55] recorded inappropriately low plasma AVP levels and an atypically sensitive pressor response to AVP infused exogenously during septic shock. The authors concluded that the deficiency in AVP contributes to the hypotension of septic shock. The levels of AVP remain exceptionally low for a period of up to 7 days following the onset of the septic shock [32]. The more serious the infection, the lower the vasopressin concentration.

#### 4.3.2. Sparing of Catecholamines/Decatecholaminization

Prolonged exposure to excessive catecholamines raises the likelihood of arrhythmias [56], critical organ damage and tissue ischemia [57,58]. Additionally, patients with higher NE requirements are at a greater risk of mortality, particularly once a threshold of 1 μg/kg/min or higher is reached, and the mortality rate can reach 90% [59], primarily due to the severity of illness but also because of the harmful effects of catecholamines.

Using early multimodal vasopressor therapy can lead to an improved safety profile, by administering drugs with diverse mechanisms at reduced doses in order to maximize safety and efficacy. In this context, AVP would act like a catecholamine-sparing drug, also limiting the immunoparalysis induced by NE (NE produces dysregulation of the immune response in mice and humans and compromises the host defenses) [60].

There may be several reasons for the enhanced sensitivity to exogenous AVP observed in septic shock:

- Adrenoceptors become less responsive or downregulated as a result of high circulating catecholamines levels [39], so vasodilation persists despite increased plasma catecholamine concentrations. Arginine vasopressin binds to V1 receptors and non-catecholamine receptors, causing vasoconstriction, and enhances vascular responsiveness to catecholamines [61].
- Arginine vasopressin can block the K-ATP channels and interfere with NO signaling, potentiating the effects of adrenergic drugs at vascular smooth muscle in shock states [62].
- AVP might also be considered in the presence of acidosis, as AVP receptor sensitivity tends to be relatively more preserved in an acidic environment when compared to adrenergic receptors [63].

The first randomized controlled trial comparing AVP to NE in septic shock (the Vasopressin in Severe Sepsis Trial [VASST]) [32] divided septic shock patients into two groups: one receiving low-dose AVP (up to 0.03 U/min) plus open-label NE, and the other receiving NE alone. At both 28 and 90 days, there were no differences in mortality rates or major organ dysfunction between the two groups. However, in patients with a lower severity of shock (receiving baseline NE up to 14 μg/min), AVP may have contributed to a nearly 10% reduction in mortality (26.5% vs. 35.7%). VASST also explored the interaction of AVP, corticosteroid and mortality in septic shock: the combination of AVP and corticosteroids resulted in lower 28-day mortality when compared to the use of corticosteroids with NE (44% vs. 35%). This study demonstrated the safety and effectiveness of AVP, while emphasizing its role in decreasing the need for NE in cases of septic shock. 

When the revised definition of septic shock was applied to patients in the VASST trial, AVP demonstrated most effective in patients with a lactate level minor than 2 mmol/L [64].

The VANISH trial [33] compared the use of AVP versus NE in septic shock and also assessed the role of hydrocortisone. There was no significant difference in terms of 28-day mortality between the AVP and NE groups. No interaction between AVP and hydrocortisone was identified.

Both the VANISH and VASST trials produced “negative” results: they did not establish the superiority of AVP over NE but showed that AVP is not inferior to NE and demonstrated a catecholamine-sparing effect of AVP. In effect, the early administration of AVP plus NE may assist in reducing the adrenergic load associated with classic vasoactive agents [65]. Furthermore, a recent evaluation revealed that higher NE doses and higher lactate concentrations at the initiation of AVP were both associated with increased in-hospital mortality [66].

The evidence supports the use of AVP as a safe vasopressor in addition to NE. The number of adverse events was similar for both drugs [67], and new-onset atrial fibrillation proved about 23% less frequent when catecholamines were combined with AVP.

However, a recent meta-analysis [68] revealed that AVP, either used alone or in combination with NE, was associated with an increased risk of digital ischemia but a lower risk of arrhythmia compared to NE alone. The risk proved higher when AVP dosing was increased without optimal fluid status and optimal cardiac output [67]. AVP would also allow the down-titration of NE in septic shock patients with left ventricular outflow tract obstruction and hemodynamic improvement, which represent 1.9% of all septic shock patients [69].

These findings suggest that it is preferable to start AVP when patients are on low NE doses or have low lactate concentrations, rather than delaying therapy.

#### 4.3.3. Nephroprotective Effect

Acute kidney injury (AKI) frequently occurs as a complication of sepsis, with a mortality rate of up to 70% [70].

**Figure 4:**
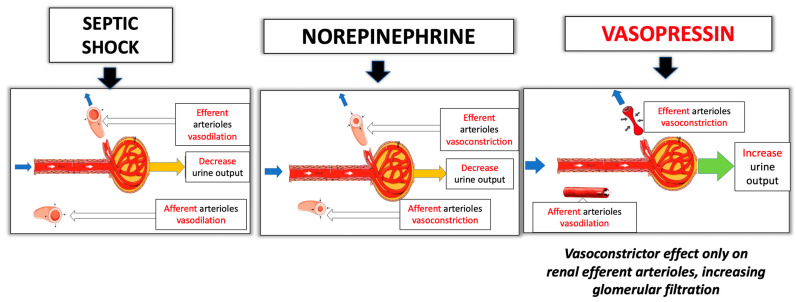
Nephroprotective effect of AVP.

AVP may maintain better renal perfusion when compared with NE due the heterogeneous distribution of the V1a receptors in the kidney (higher concentration of V1 receptors in glomerular efferent than in afferent arterioles) [71]: the vasoconstrictor effect of AVP acts predominantly on the renal efferent arterioles, with minimal action on the afferent arterioles, increasing glomerular filtration [13] (Figure 4). In small clinical studies, the infusion of AVP has been shown to increase urine output and improve creatine clearance when compared to NE [49,72].

Gordon et al. [73] conducted a post hoc analysis of the VASST to assess the influence of AVP on AKI, and they found that among patients classified in the Risk category of the RIFLE scoring system [74], a significantly lower percentage of those treated with AVP (21% vs. 40%) advanced to the Failure or Loss categories or required dialysis (17% vs. 38%). In the VANISH trial [33], although there were no differences with respect to kidney injury, the use of AVP reduced the probability of requiring renal replacement therapy (RRT). In another recent meta-analysis [75] that compared AVP with catecholamines vs. catecholamines alone, it was found that the incidence of AKI and the requirement for RRT were reduced with the use of catecholamines with AVP.

#### 4.3.4. Early onset of AVP Infusion

Therapy with AVP preferably should be started within the first 3–6 h after the onset of septic shock. It is clear that earlier vasopressor initiation is better than later initiation, but the timing of a secondary agent is less clear [76,77]. There is increasing evidence that early AVP infusion improves the prognosis of patients with septic shock. In VASST [32], a tendency indicated lower mortality with the use of AVP. A single-center, prospective open-label trial of early AVP in the first four hours of NE showed faster achievement and maintenance of the target mean arterial pressure (MAP) compared to NE monotherapy [78]. According to a recent study examining over 1500 patients with septic shock, the probability of death during hospital admission increased by 20.7% for each 10 µg/min rise in NE dosage when AVP was introduced as second-line treatment [66].

These findings indicate that starting AVP early could be helpful and should be used to target the hemodynamic response for ongoing treatment.

### 4.4. Interactions of AVP and Corticosteroid Treatment in Septic Shock

Corticosteroids have been used for many years in patients with septic shock who are undergoing vasopressor treatment, and recent SSC guidelines have also established these recommendations [21].

A further post hoc analysis of the VASST trial [32] revealed that low-dose AVP in combination with corticosteroids resulted in reduction in 28-day mortality compared to corticosteroids with NE (44.7% to 35.9%). Conversely, in cases where patients were not treated with corticosteroids, AVP might have led to an increase in mortality compared with NE. A trend was observed in the trials in that severely ill septic shock patients with very high doses of catecholamines benefited more than patients treated with lower doses of catecholamines [71,79].

The mechanism through which AVP plus corticosteroids leads to reduced mortality remains unclear. Possible hypotheses include that corticosteroids may elevate the AVP levels, could improve responsiveness to AVP [80] and would benefit inflammation and immunity [81]. Arginine vasopressin activates the hypothalamus–pituitary–adrenal axis, releasing adrenocorticotropic hormone (ACTH) and increasing serum cortisol by binding to V3 receptors. Other authors [82] suggest that interactions with nitric oxide could explain this phenomenon.

### 4.5. Personal Recommendations for the Use of AVP in Septic Shock in Clinical Practice

The existence of various intravenous NE formulations (salt and base) is not widely known [83]. The salt formulation guarantees drug stability and solubility, while the base formulation is pure NE. Surprisingly, NE base is not commercially available anywhere in the world.

The predominant commercial NE preparations are NE tartrate and bitartrate. These salts are half as potent as NE base, with 1 mg of NE tartrate/bitartrate being equivalent to 0.5 mg of NE base (NE tartrate doses are twice as high as those expressed as NE base) [84]. The NE formulation referred to by the SSC guidelines is NE base; thus, if the SSC recommends adding AVP starting from an NE base dose of 0.25–0.5 μg/kg/min, it would be equivalent to an NE tartrate dose of 0.5–1 μg/kg/min. In this review, we will refer to the doses of NE base. Nevertheless, an international consensus is needed regarding the formulation used to describe the NE dose in order to avoid dosing confusion in practice and to facilitate the interpretation of clinical trials. Furthermore, it would be advantageous for the NE salt formulations available to clearly indicate the equivalent dose of NE base on their labels [85].

The NE-equivalent dose can be used to determine when to initiate AVP. If septic shock patients prove unresponsive to low doses of NE and require higher doses of up to 0.25 μg/kg/min, it is essential to investigate the reason for their lack of response before starting a second vasopressor, ruling out concomitant cardiac dysfunction or any reversible metabolic abnormalities. Once such causes have been ruled out, for patients requiring doses of 0.25 μg/kg/min or higher, the addition of a second vasopressor may be advantageous.

Clear definitions of refractory vasodilatory shock are lacking, but a threshold of 0.5 μg/kg/min is generally accepted. Although the adequate timing of initiation of a second-line vasopressor remains a challenge, it might be a good option to add AVP earlier when the NE dose is in the range of 0.25–0.5 μg/kg/min [21,33], as recommended by the SSC guidelines, and not wait until refractory shock is established.

**Figure 5:**
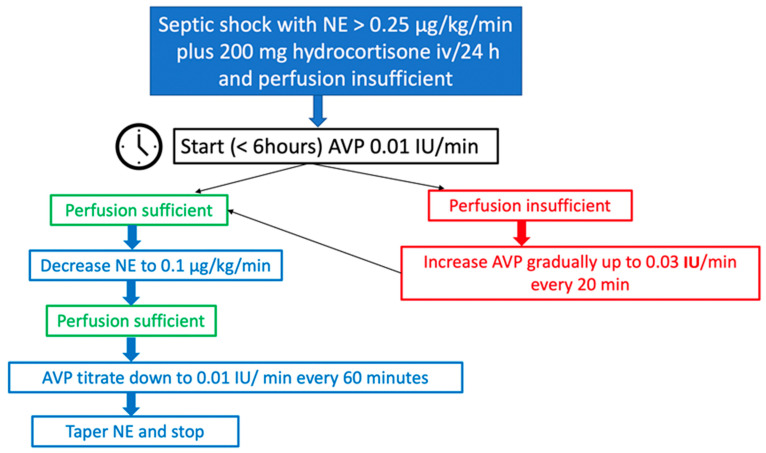
Our AVP protocol in septic shock. IU: international unit; min: minute; h: hour; AVP: arginine vasopressin; NE: norepinephrine; iv: intravenous.

Our approach to the use of AVP in septic shock (Figure 5) includes the early introduction of multimodal vasopressors, also termed “broad-spectrum vasopressors” [86,87]: in patients where the blood pressure or perfusion goals are not reached despite NE 0.25 μg/kg/min, adequate fluid resuscitation, added corticosteroids and adequate high cardiac output, AVP can be started with 0.01 IU/min and slowly increased to 0.03 IU/min in steps of 20 min. When the target blood pressure is achieved and sufficiently maintained, the NE infusion should be slowly decreased to 0.1 μg/kg/min, after which AVP should be down-titrated 0.01 IU/min every 60 min, provided that the blood pressure is stable. Surprisingly, a recent survey revealed that roughly 70% of clinicians discontinue AVP without gradual titration [88], and some studies support this practice [89], though we prefer gradual reduction of the drug.

Once AVP infusion is discontinued, NE should be tapered till stopped. Among clinicians, there is no consensus on the best method for stopping AVP in patients recovering from septic shock, and it is unclear which vasopressor should be discontinued first (NE or AVP). In our practice, we stop AVP first, because NE is still the first-choice vasopressor. However, some studies show that tapering NE before AVP implies a lesser risk of rebound hypotension [90,91,92].

The proposed dosing regimen for septic shock (0.01–0.03 IU/min) is consistent with the dose used in the pivotal clinical trial (VASST). The choice of dose is reasonable considering the possible lack of additional efficacy at doses higher than 0.04 IU/min and the potential risk of adverse events [48,93]. The response to AVP could be an early indicator of the patient prognosis (improved clinical course), and additional assessments should be made in AVP non-responders (e.g., echocardiography), possibly combined with changes in therapy [94].

Researchers have recently explored the cost-effectiveness of second-line vasopressors through a comparison of escalating NE doses versus the use of NE in combination with adjunctive AVP or angiotensin II for septic shock. In this regard, AVP proves to be the most cost-effective second-line vasopressor, though vasopressor price plays a minor role in overall cost [95]. It is recommended to consider administering epinephrine as second-line therapy in patients exhibiting inadequate cardiac output (mixed cardiogenic shock) or an inappropriately low heart rate, due to its beta-receptor action and lack of inotropic support with AVP [58]. If neither of these drugs prove effective, angiotensin II may be used, particularly if the patient requires an NE dose exceeding 0.25 μg/kg/min after the addition of AVP.

The recommended AVP doses are independent of body weight. Administering a fixed dose regardless of body weight may predispose patients to either increased toxicity risk or decreased efficacy [96]. There are conflicting data on the influence of body weight on AVP response, and based on available evidence, it is advisable not to exceed the established AVP doses in obese patients [97,98].

### 4.6. Use of Higher Doses of AVP in Septic Shock?

The VANISH trial employed twice the dosage of AVP used in VASST (0.01–0.06 U/min versus 0.01–0.03 U/min), with the hypothesis that a higher dose may confer a survival advantage not seen in the first trial. However, the VANISH trial did not reveal any such benefit despite the increased AVP dosage, and it reported a higher incidence of digital ischemia in the AVP group (5% vs. 1.5%). Several studies have investigated vasopressin doses of approximately 0.06 U/min (as opposed to 0.03 U/min in VASST) and have found this higher dose to be more effective in reversing cardiovascular failure in vasodilatory shock [93,99] but with more adverse effects (like intestinal ischemia, increased cytolysis and cholestasis, and thrombocytopenia). A large, randomized trial of these higher vasopressin doses (0.06 U/min) would be needed to address safety and efficacy issues in septic shock.

### 4.7. Future Directions

The future of sepsis treatment will focus on the definition of endotypes (subtypes characterized by a distinct biological mechanism linking clinical characteristics with a concrete molecular pathway) leading us to personalized therapy [100] in which patients receive the right medicine for them.

In order to tailor vasopressor therapy for vasodilatory shock, it is necessary to establish a role for biomarker-guided non-catecholamine vasopressor initiation to personalize resuscitation [87] and improve patient selection for therapy: how to identify vasopressor responders and patients that may be less reactive to one vasopressor but more reactive to another due to differences in host genotype, variable organ-specific receptor expression and downregulation in different tissues. There is currently no bedside test for predicting blood pressure response to catecholamines, AVP or angiotensin II, though several emerging candidate biomarkers have shown correlations to vasopressor response and outcomes, such as:

- Genetic variations in ARDβ2, which encodes the β2-adrenergic receptor, have been linked to higher mortality rates and greater organ dysfunction in septic shock (with greater NE requirements, increased renal, hematological, hepatic and neurologic dysfunction, and increased 28-day mortality) [101].
- Genetic variations in LNPEP (leucyl and cystinyl aminopeptidase), also known as vasopressinase, have been associated with higher plasma clearance of AVP, serum sodium regulation and increased 28-day mortality [102].
- It has been proposed that plasma AVP levels could serve as a guide for AVP therapy, aiming to target physiological levels while avoiding higher concentrations that may lead to adverse effects [103]. Nevertheless, the ideal serum AVP concentration for septic shock remains uncertain. Measuring plasma AVP levels presents challenges due to its short half-life and ex vivo instability. On the other hand, the copeptin, i.e., the C-terminal of AVP precursor, is stable in plasma, easier to assay than AVP and exhibits a strong correlation with AVP plasma concentration. Therefore, future studies are necessary to assess the potential utility of copeptin levels for guiding AVP therapy [81].
- Serum renin is emerging as a predictor of mortality; it seems to remain stable and unaffected by RRT or drugs [104]. The administration of exogenous angiotensin II has demonstrated a beneficial impact on survival outcomes in individuals with high-renin shock [105].

The application of machine learning for the early identification and improved selection of vasopressors has the potential to improve outcomes.

## 5. AVP in Vasodilatory Shock in Heart Surgery

Vasodilatory shock in heart surgery is a well-recognized syndrome occurring in 9–44% of all patients undergoing cardiopulmonary bypass (CPB) procedures, with an associated mortality rate of up to 25% [106]. It is also known as vasoplegic syndrome, and like the early stages of septic shock, it is characterized by low mean arterial pressure (<60–65 mmHg), markedly low systemic vascular resistance, a normal or elevated cardiac index and a poor or insufficient response to fluid or catecholamine administration. Among other risk factors (Table 4), vasoplegic syndrome occurs more frequently in populations with congenital heart disease undergoing heart surgery and in patients with heart failure requiring the implantation of a ventricular assist device or heart transplant [107,108].

### 5.1. Pathophysiology of Vasoplegic Shock in Heart Surgery

Activation of the intrinsic vasodilatory and coagulation pathways occurs with the continuous exposure of blood to foreign surfaces of the cardiopulmonary circuit. These include the contact system, the extrinsic and intrinsic coagulation pathways, the complement system and fibrinolysis. As a result of contact system activation, an increased release of bradykinin and kallikrein takes place. Activation of the complement system in turn results in C5a and terminal complement complex formation. In consequence, leukocytes, platelets, macrophages and neutrophils are also activated. Such a cascade results in systemic inflammatory response syndrome (SIRS), with the release of multiple cytokines and proinflammatory and prothrombotic substances, endothelial cell activation and the secretion of vasoactive substances including NO and prostacyclin [109,110].

In addition, in order to reduce blood loss from the surgical field, blood is conventionally suctioned into the extracorporeal pump, filtered and re-infused into the body. Such retrieved blood is loaded with inflammatory mediators produced locally as a result of surgical trauma, contributing to the generalized inflammatory response accompanying vasoplegic syndrome [111].

Regarding vasopressin and vasoplegic shock, patients undergoing heart surgery who present vasodilatory shock have shown significantly lower levels of AVP when compared to those who did not present vasodilatory shock, mainly in the first 48 h after surgery [112]. It has been recognized that blood levels of AVP and copeptin (a glycoprotein needed for the synthesis of a pre-provasopressin precursor in the hypothalamus) are chronically elevated in patients with heart failure and reduced ejection fraction, which could explain the depletion of AVP seen in patients exposed to the hemodynamic stress of heart surgery [113,114].

Perioperative vasopressin deficiency has been demonstrated in patients undergoing ventricular assist device implantation who more frequently present vasoplegic syndrome. Argenziano et al. [115] analyzed the AVP levels in patients undergoing LVAD implantation, finding levels between 3.6 and 6.3 pg/mL, which are well below the usual levels in cardiopulmonary bypass (100–200 pg/mL). The theoretical explanation is autonomic failure associated with the depletion of neural storage. Patients who underwent vasoconstrictor therapy with AVP presented a significant increase in mean arterial pressure and systemic vascular resistances within 15 min of the start of administration. Patients with greater AVP deficiency were those with the most significant response.

### 5.2. Rationale for AVP Use in Heart Surgery

Multiple observational studies and randomized controlled trials (RCTs) of AVP versus a placebo or catecholamines have been carried out in the context of heart surgery. Dünser et al. [116] published a meta-analysis of eight RCTs involving 625 patients undergoing heart surgery and on CPB, with procedures that included coronary artery disease surgery and valvular and/or aortic surgery. The results suggested that the AVP use reduces the occurrence of perioperative complications, mainly due to a decrease in the incidence of vasodilatory shock and the onset of new atrial fibrillation. Arginine vasopressin significantly increases MAP, allowing a significant reduction of the NE doses, when used concomitantly, without increasing mortality or mesenteric or digital ischemia. 

The VANCS study included 330 patients randomized to AVP (0.01–0.06 U/min) versus NE (10–60 µg/min). This study showed a decrease in the primary outcome of mortality or complications, mainly due to a reduction of renal failure (32.2% vs. 49%; *p* = 0.0014), as well as a lower incidence of atrial fibrillation at 30 days (63.8% vs. 82.1%; *p* = 0.0004) [117]. Okamoto et al. randomized patients to NE versus NE plus AVP and recorded a decrease in the incidence of tachycardia (53.3% vs. 74.5%; *p* = 0.03) and postoperative atrial fibrillation (36.2% vs. 64.9%; *p* = 0.0489) in the AVP group [118]. More importantly, no greater digital or mesenteric ischemia was evidenced in the groups exposed to AVP when compared to NE. Another study by Dünser et al. randomized patients to NE versus NE + AVP at a dose of 0.06 U/min, achieving a significantly higher MAP in the AVP group, and with a lower incidence of tachyarrhythmias (8.3% vs. 54.3%) [50]. 

With regard to dosing, infusions of 0.01 U/min correlate to plasma levels <40 pg/mL, corresponding to physiological values. Higher doses of up to 0.1 U/min are adequately tolerated and correspond to plasma levels of around 150 pg/mL [108].

On addressing the complications related to high dose AVP, one of the risks described is renal failure. A retrospective study of 280 patients suggested a dose-dependent relationship, although it appears only extraordinarily when the doses used are under 0.04 U/min [119]. Another reported toxic effect is digital or mesenteric ischemia, which has been demonstrated in porcine models at doses above 0.4 U/min [120]. Myocardial ischemia has been studied by measuring biomarkers of myocardial damage 12 h after heart surgery, with no differences being found when comparing NE versus AVP [118]. In general, the recommendation is to administer a dose lower than 0.1 U/min to reduce the likelihood of side effects.

Experts in heart surgery and cardiovascular intensive care have issued a consensus statement, giving a strong recommendation to starting NE and/or vasopressin in order to maintain systemic perfusion pressure. There is a strong recommendation to initiate vasopressin early in the presence of NE side effects due to an excess adrenergic response or tachyarrhythmias [121]. As standard practice in heart surgery, it is advisable to initiate vasopressin when the doses of NE are above 0.2 µg/kg/min [122].

### 5.3. Personal Recommendations for the Use of AVP in Vasoplegic Syndrome in Heart Surgery (Figure 6)

#### 5.3.1. Preoperative Period

We recommend systematic preoperative risk evaluation for vasoplegic syndrome and post-cardiotomy cardiogenic shock in order to adjust invasive monitoring. In high-risk patients (heart transplant, LVAD, low ejection fraction, etc.), we recommend the femoral artery instead of the radial artery for invasive arterial pressure measurement. In such high-risk patients, we advise adding a pulmonary artery catheter (PAC) from the start of the intervention.

**Figure 6:**
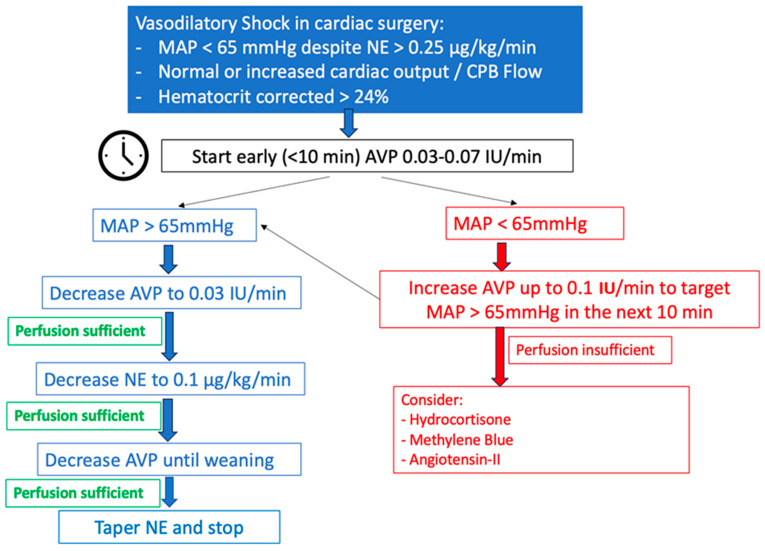
Our AVP protocol in heart surgery.

#### 5.3.2. Intra- and Postoperative Period

Once on CPB, if MAP is below 65 mmHg despite adequate blood output, NE should be started and rapidly titrated to reach this threshold. Factors such as severe hemodilution after starting CPB should be recognized and treated as well [123]. As mentioned earlier in the text, we will refer to NE dosage as a formulation of NE base. Vasopressin infusion should be initiated if MAP is persistently below 65 mmHg despite NE > 0.25 μg/kg/min and a hematocrit of over 24%. We normally start AVP at 0.03–0.07 IU/min, depending on the depth of the hypotensive episode, with rapid titration until MAP 60 mmHg is achieved within 10 min. Once MAP is above 75 mmHg, we start decreasing the AVP dose to maintain MAP 65–75 mmHg during CPB. We try not to increase AVP beyond 0.1 IU/min in order to reduce adverse reactions such as renal failure, but in some cases and for a brief time, we have needed to use doses beyond such a limit without having immediate or postoperative complications such as digital or mesenteric ischemia.

After the aortic cross-clamp is released and separation from cardiopulmonary bypass occurs, we recommend adjusting inotropes or vasopressors following measurements of systemic vascular resistance and cardiac output when PAC is available. If not, using indirect measures of cardiac output and direct measures of ventricular function with transesophageal echocardiography appears reasonable.

#### 5.3.3. Weaning from AVP

There is no general consensus or recommendation for weaning from AVP in heart surgery. At our institution, If MAP is maintained above 65 mmHg during the post-CPB period, either in the operating room or in the ICU, the AVP dose is lowered to 0.03 IU/min. Then, if hemodynamic stability allows it, NE is tapered down to 0.1 μg/kg/min. Finally, AVP is weaned off first.

## 6. AVP in Cardiac Arrest

Historically, both epinephrine and AVP have been used in the context of circulatory arrest. However, the present evidence shows no benefit in using AVP in this setting, and the current international resuscitation guidelines do not recommend AVP as vasopressor therapy [124].

A systematic review of vasopressors in adult cardiac arrest was conducted by the International Liaison Committee on Resuscitation Advanced Life Support Task Force and published in 2019. Three RCTs comparing AVP versus epinephrine during out-of-hospital cardiac arrest (OHCA) were included. The results showed no significant differences between the groups in terms of return to spontaneous circulation (ROSC), survival to hospital admission, survival to hospital discharge or survival to hospital discharge with favorable neurological outcome. Subgroup analysis based on initial rhythm also did not reveal statistically significant differences. Additionally, this review included three RCTs comparing the combined use of epinephrine and AVP to epinephrine alone during OHCA, and no significant differences were found in terms of ROSC, survival to hospital admission or survival to discharge [125].

At present, epinephrine is the only recommended vasopressor during cardiac arrest, with a strong grade of recommendation both in situations of shockable rhythm after an unsuccessful first shock and in situations of non-shockable rhythms. There are recommendations against the administration of AVP instead of or in addition to epinephrine during cardiac arrest [124].

## 7. Authorized Indications and Dosages in Europe and the United States of America (USA)

The European Medicines Agency (EMA) authorized AVP for the management of catecholamine refractory hypotension following septic shock in patients older than 18 years. AVP should be administered through continuous intravenous infusion of 0.01 IU per minute using a motor pump. Dependent on the clinical response, the dose may be increased every 15–20 min up to a maximum of 0.03 IU/min. 

On the other hand, the USA authorized AVP for vasodilatory shock in adults (sepsis and post-cardiotomy); for septic shock, it recommends starting with a dose of 0.01 IU/min (maximum 0.07 IU/min), and for post-cardiotomy shock, starting with a dose of 0.03 units/minute (maximum 0.1 IU/min).

## 8. Adverse Effects of AVP

Due to its potent vasoconstrictor effect, there have been concerns about the potential impact of AVP on splanchnic circulation, with a fear of splanchnic ischemia and liver dysfunction [13]. Experimental studies have refuted these concerns, demonstrating that with adequate fluid resuscitation, mesenteric blood flow and ileal microcirculation remain preserved [126]. Furthermore, clinical trials have not reported any adverse effects upon splanchnic circulation with the use of AVP [126].

The rate of serious adverse effects was comparable between the AVP and NE groups in the VASST study [81]. However, there were more cardiac arrests in patients in the NE group and higher occurrence of digital ischemia and hyponatremia with the AVP group. Other side effects of both AVP and NE include reduced cardiac output, skin necrosis and intestinal ischemia.

AVP induces vasoconstriction in cutaneous blood vessels, and this effect is dose-dependent. A retrospective study found that nearly one-third of patients exposed to AP experienced ischemic skin lesions [127]. Risk factors associated with the development of ischemic cutaneous lesions included being overweight, receiving a high dose of NE, receiving platelets and fresh frozen plasma transfusions, having a history of peripheral arterial occlusive disease and the occurrence of septic shock. After conducting a multivariate analysis, only the latter two factors remained associated with the occurrence of cutaneous complications.

## 9. Conclusions

Septic shock is a complex disorder associated with high mortality. High-dose norepinephrine as monotherapy may not be the best approach. Vasopressin is a second-line vasopressor option and may be added early to norepinephrine to achieve the following targets: catecholamine-sparing effects (fewer adverse events in relation to NE) and nephroprotective effects. We should not wait until refractory shock occurs.

Arginine vasopressin has demonstrated reliable results in vasodilatory shock related to heart surgery. It should be initiated early as hypotension persists despite fluid resuscitation, hematocrit correction and standard NE infusion. Although high doses may be necessary in the short term to restore organ perfusion pressure, the catecholamine-sparing effect seen when adding AVP could explain the reduced incidence of atrial fibrillation and other frequent tachyarrhythmias, with no more frequent limb or mesenteric ischemia when compared to isolated high-dose NE.

In the context of cardiac arrest, AVP has not been demonstrated to be beneficial, and the current guidelines advise against its use.

In the near future, it will be desirable to have bedside tests to predict which patients will respond to a specific vasopressor and to identify the patient populations that would benefit most from AVP use.

## Figures and Tables

**Table 1:** Vasopressin agonists.

VASOPRESSIN AGONISTS	STRUCTURE	RECEPTOR AFFINITY	CLINICAL APPLICATION	HALF-LIFE (min)
ARGININE VASOPRESSIN (AVP)	8-Arginine vasopressin	V1, V2, V3	Sepsis, vasodilatory shock, cardiac arrest	5–15
DESMOPRESSIN ACETATE (DDAVP)	Deamino-Cys-D-Arg vasopressin	V2	Central diabetes insipidus, bleeding disorders	90–190
TERLIPRESSIN (TP)	N3-triglycyl-8-lysin vasopressin	V1	Portal hypertension, bleeding gastric and esophageal varices, septic shock	240–360
SELEPRESSIN	Phe-2-Ile-3-Hgn-4-Orn-8 vasopressin	V1	Septic shock Not approved for clinical use	10–30
ORNIPRESSIN	8-L-Ornithine vasopressin acetate	V1	Vasoconstricting agent during myomectomy in cirrhosis, as hepatorenal treatment	60–120

**Table 2:** Pivotal trials of vasopressors in septic shock.

TRIAL	Intervention	Control	Intervention 28 Day Mortality	Control 28 Day Mortality	Absolute Difference (95%CI) *p*-Value
VASST [32]	Norepinephrine	Vasopressin	35.4%	39.3%	3.9 (−2.9–10.7) 0.26
VANISH [33]	Norepinephrine	Vasopressin	30.9%	27.5%	3.4 (−5.4–12.3)
SOAP II [34]	Norepinephrine	Dopamine	48.5%	52.5%	1.17 (0.97–1.42) 0.10
ATHOS-3 [35]	Angiotensin II	Placebo	46%	54%	Hazard ratio 0.78 (0.57–1.07) 0.12
CAT [36]	Epinephrine	Norepinephrine	23%	27%	Hazard ratio 0.87 (0.48–1.58) 0.65
CATS [37]	Epinephrine	Norepinephrine + dobutamine	40%	34%	Relative risk 0.86 (0.65–1.14) 0.31

**Table 3:** Vasopressors. SNP: single nucleotide polymorphism; LNPEP: leucyl cystinyl aminopeptidase; AGTRAP: angiotensin II receptor associated protein; GABAA: gamma-aminobutyric acid type A.

Vasopressor	Receptor Activity	Vasa Constriction	Inotropism	Doses	Possible Predictive Biomarkers
Norepinephrine	α1 > β1, β2	++++	++	0.04–1 μg/kg/min	β2 receptor SNP
Vasopressin	V1, V2, V3	++++	0	0.01–0.03 IU/min	LNPEP SNP Angiopoietin 1/2 Vasopressin/copept in
Epinephrine	β1 > α1, β2	++++	++++	0.01–0.1 μg/kg/min (β) >0.1 μg/kg/min (α)	β2 receptor SNP
Dopamine	D1, α1, β1	++-+++	++-+++	Inotropic: 5–10 μg/kg/min Vasopressor > 10 μg/kg/min	
Phenylephrine	α1	++++	0	0.1–1.5 μg/kg/min	
Angiotensin-ll	AngiotensinⅡ receptors	++++	0	5–200 ng/kg/min	AGTRAP SNP
Methylene blue	Inhibits GABAA receptors	++++	0	Bolus(2 mg/kg) then infusion: 0.25–1.2 mg/kg/h	

**Table 4:** Risk factors associated with vasoplegia in heart surgery.

Predominant and independent risk factors	- Type of procedure (OHT, LVAD, CHD, combined surgery) - Reduced ejection fraction < 35% - Thyroid disease - VAD before surgery - Preoperative use of intravenous heparin, ACEIs or beta-blockers
Other risk factors	- Duration of CPB - Higher body mass index - Pre-existing endothelial cell activation reflected by higher baseline von Willebrand factor propeptide and sP-selectin levels - High preoperative plasma copeptin concentration - Low AVP concentration - Increased adenosine levels - Ischemia-modified albumin

OHT: orthotopic heart transplant; LVAD: left ventricular assist device; CHD: congenital heart disease; VAD: ventricular assist device; ACEIs: angiotensin-converting enzyme inhibitors; CPB: cardiopulmonary bypass; AVP: arginine vasopressin. Adapted from Omar et al. [102].
